# In situ analysis of neuronal injury and neuroinflammation during HIV-1 infection

**DOI:** 10.1186/s12977-024-00644-z

**Published:** 2024-07-01

**Authors:** Jenna B. Honeycutt, Angela Wahl, Jacob K. Files, Alexis F. League, Barkha J. Yadav-Samudrala, J. Victor Garcia, Sylvia Fitting

**Affiliations:** 1 Division of Infectious Diseases, Center for AIDS Research, School of Medicine, University of North Carolina at Chapel Hill, Chapel Hill, NC 27599 USA; 2 Department of Microbiology, University of Alabama at Birmingham, Birmingham, Alabama, AL 35294 USA; 3 Department of Psychology & Neuroscience, University of North Carolina at Chapel Hill, Chapel Hill, NC 27599 USA

**Keywords:** Humanized mice, CCR5-tropic virus isolates, CH040, JR-CSF, CD68, Astrocytes, Microglia, Neuronal injury, Frontal cortex, Striatum

## Abstract

**Background:**

Since the introduction of combination antiretroviral therapy (cART) the brain has become an important human immunodeficiency virus (HIV) reservoir due to the relatively low penetration of many drugs utilized in cART into the central nervous system (CNS). Given the inherent limitations of directly assessing acute HIV infection in the brains of people living with HIV (PLWH), animal models, such as humanized mouse models, offer the most effective means of studying the effects of different viral strains and their impact on HIV infection in the CNS. To evaluate CNS pathology during HIV-1 infection in the humanized bone marrow/liver/thymus (BLT) mouse model, a histological analysis was conducted on five CNS regions, including the frontal cortex, hippocampus, striatum, cerebellum, and spinal cord, to delineate the neuronal (MAP2ab, NeuN) and neuroinflammatory (GFAP, Iba-1) changes induced by two viral strains after 2 weeks and 8 weeks post-infection.

**Results:**

Findings reveal HIV-infected human cells in the brain of HIV-infected BLT mice, demonstrating HIV neuroinvasion. Further, both viral strains, HIV-1_JR-CSF_ and HIV-1_CH040_, induced neuronal injury and astrogliosis across all CNS regions following HIV infection at both time points, as demonstrated by decreases in MAP2ab and increases in GFAP fluorescence signal, respectively. Importantly, infection with HIV-1_JR-CSF_ had more prominent effects on neuronal health in specific CNS regions compared to HIV-1_CH040_ infection, with decreasing number of NeuN^+^ neurons, specifically in the frontal cortex. On the other hand, infection with HIV-1_CH040_ demonstrated more prominent effects on neuroinflammation, assessed by an increase in GFAP signal and/or an increase in number of Iba-1^+^ microglia, across CNS regions.

**Conclusion:**

These findings demonstrate that CNS pathology is widespread during acute HIV infection. However, neuronal loss and the magnitude of neuroinflammation in the CNS is strain dependent indicating that strains of HIV cause differential CNS pathologies.

**Supplementary Information:**

The online version contains supplementary material available at 10.1186/s12977-024-00644-z.

## Background

Combination antiretroviral therapy (cART) has significantly improved the prognosis for people living with human immunodeficiency virus type 1 (HIV-1, PLWH), leading to reduced mortality rates and increased life expectancy [1–3]. However, the damage caused to the central nervous system (CNS) by HIV during infection has been difficult to establish because of the intrinsic difficulties associated with the limited access to the CNS. Furthermore, despite the effectiveness of cART in suppressing viral replication, virus replication/gene expression appears to persist at low levels, resulting in chronic immune activation and ongoing viral replication, specifically in the brain where cART penetration is limited due to the presence of the blood-brain-barrier (BBB) [4–6]. The establishment of the CNS as a HIV reservoir is highlighted by findings of virally suppressed PLWH with undetectable virus load in the blood, but detectable virus levels in the cerebrospinal fluid (CSF) [7–10] or viral DNA in post-mortem human brain tissue [11, 12]. More importantly, low-level productive viral infection in the CNS contributes to brain volume changes and cognitive impairments in virally suppressed PLWH on cART compared to uninfected individuals [13–16].

HIV entry from the periphery into the CNS can occur within less than two weeks of infection [17, 18], via cell free virus [19], through infected CD4^+^ T cells [20–22], or infected monocytes that migrate to the CNS and differentiate into macrophages [23, 24], all of which allow HIV to disseminate into the CNS [25, 26]. Even though CD4^+^ T cells are the preferred target for HIV cell entry [27], microglia and perivascular macrophages are also important target cells within the CNS [26, 28]. HIV can replicate within the CNS independently of the rest of the body due to viral compartmentalization [25] and can potentially evolve over time within an individual from a T cell-tropic virus upon initial infection to a macrophage-tropic variant in the CNS/CSF [17, 21, 29]. Nevertheless, a subtype of CD4^+^ T cells, known as the CD4^dim^CD8^bright^ T cells, has been recently found to resist HIV-mediated cytopathy [22, 30], and was detected in the CSF of PLWH [31], thus potentially playing a significant role in maintaining CNS infection.

While human studies have played a crucial role in enhancing our understanding of HIV effects on the CNS, the systematic investigation of specific viral variants and their effects on the CNS, including the timing of infection and strain, is limited due to the use of CSF or post-mortem tissue samples that are variable in timing of infection, viral load, viral strain, and immune cell expression [32]. Hence, to gain a better understanding of the underlying pathophysiological mechanisms involved in HIV CNS infection the use of animal models is essential to study specific aspects of the disease and to test new therapeutic approaches for “HIV cure” [32]. Specifically, humanized mice, have been extensively utilized to investigate fundamental questions regarding immunopathogenesis of HIV infection, including HIV neuroinvasion, CNS viral replication, and HIV-associated CNS pathology [33–38].

The assessment of HIV-induced pathologies on the CNS and related cognitive deficits in HIV infected humanized mice has been studied to a certain extent [39–41]. Dendritic injuries, astrogliosis and microgliosis, as well as learning and memory deficits have been reported in the SCID-HIVE mouse model [42–44], in which human monocyte-derived macrophages that are infected with the laboratory-adapted macrophage-tropic R5 strain, HIV-1_ADA_, are injected directly into the frontal lobe [45, 46]. Nevertheless, due to the nature of injecting virus infected cells directly into the SCID-HIVE mouse brain it is less translatable to the human HIV condition. Another model that mimics ongoing viral infection and HIV neuroinvasion is the humanized NOD/SCID-IL-2Rγ_c_^nul^ (hNSG) mouse model in which human cells, either peripheral blood mononuclear cells (PBMCs) or CD34^+^ hematopoietic stem cells, are transplanted and subsequently infected with HIV via injection [47–49]. In this model, HIV-induced CNS pathology was more subtle but included deficits in neuronal integrity and increases in astrocytes and microglia in specific brain regions, such as the cortex [47–49], with infection by HIV-1_ADA_ demonstrating increased anxiety and locomotor-related memory deficits [48]. Besides these mentioned models no other humanized mouse model to date has focused on CNS pathological changes upon HIV neuroinvasion, including the potentially unique contribution of different virus strains, and how their effects change over the time course of infection.

We recently performed a systematic study to characterize the cellular and viral changes that occur in the CNS of bone marrow/liver/thymus (BLT) humanized mice during HIV infection [34]. A unique feature of the model is that it is systemically reconstituted with human immune cells including T cells, myeloid cells, B cells, and NK cells, with the human T cells being generated in the context of a fully functional human thymus [34, 39]. The presence of human hematopoietic cells, including T cells, B cells, and macrophages, has been demonstrated in various brain regions of BLT mice as well as persistent HIV infection in the brain [34, 50, 51]. Specifically, two CCR5-tropic strains HIV-1_JR − CSF_, a HIV early passage virus isolated from CSF, and HIV-1_CH040,_ a transmitted/founder strain, were shown to establish HIV infection in the brains of BLT mice, in particular the midbrain, medulla, thalamus, and cerebellum [34]. Further, ART efficiently suppressed HIV RNA and DNA levels in the brain and restored CD4^+^ T cells to levels of naive mice [34]. Despite these findings, the contribution of HIV to CNS pathology, including effects on neuronal integrity and neuroinflammation, in BLT mice over the course of infection is currently not known.

Thus, the goal of the present study was to assess the changes occurring in the CNS by two different strains during HIV infection using BLT humanized mice. Specifically, we were interested in delineating the neuronal and neuroinflammatory changes induced by HIV-1_JR − CSF_ and HIV-1_CH040_ on the frontal cortex, hippocampus, striatum, cerebellum, and spinal cord. Our results indicate that both viral strains induced CNS changes as early as 2 weeks post-infection, including neuronal injury and astrogliosis, across all CNS regions, with CNS-region- and viral strain-specific effects noted for neuronal loss and microglia presence. Whereas HIV-1_JR − CSF_ induced more deleterious effects on neuronal health in specific CNS regions, including the frontal cortex, HIV-1_CH040_ demonstrated more prominent effects on neuroinflammation across CNS regions, especially at 2 weeks post-infection. Our results demonstrate the differential effects of two HIV-1 strains on CNS pathology implying that different HIV strains can cause different types of disease.

## Methods

### Generation of humanized mice

Humanized mice were constructed as previously described using 11-14-week-old male and female NOD.Cg-Prkdcscid ll2rgtm1Wjl/SzJ mice (NSG mice; The Jackson Laboratory, *N* = 16) [34, 52–59]. Human immune cell levels were monitored longitudinally in the peripheral blood of mice using flow cytometry [34, 52–59]. Mice were kept on a 12 h light/dark cycle and housed in a temperature and humidity-controlled vivarium and maintained under specific-pathogen free conditions by the Division of Comparative Medicine at the University of North Carolina at Chapel Hill. All research procedures were conducted in strict accordance with the guidelines outlined in the NIH Guide for the Care and Use of Laboratory Animals (NIH Publication No. 85−23) and approved by the Institutional Animal Care and Use Committee (IACUC) at the University of North Carolina at Chapel Hill.

### Flow cytometric analysis to assess peripheral blood humanization

Human immune cell levels in peripheral blood were analyzed by flow cytometry prior to HIV-1 exposure as previously described using the following antibody panel: hCD45-APC (clone HI30, BD Biosciences), hCD19-PE (clone HIB19, BD Biosciences), hCD3-FITC (clone HIT3a, BD Biosciences), hCD4-PerCP (clone SK3, BD Biosciences) [34, 52–59]. Whole blood was incubated with purified mouse IgG (15 µg) for 5 min at room temperature (RT) to block Ig binding sites prior to antibody staining for 20 min at RT. Next, red blood cells were lysed with 1x BD FACS Lysing Solution (BD Biosciences) and then cells were washed and fixed with 2% paraformaldehyde fixative. Data was acquired on a BD FACSCanto instrument and analyzed with BD FACSDIVA software.

### HIV-1 exposure and HIV RNA analysis

Stocks of HIV-1_JR − CSF_ and HIV-1_CH040_ were prepared by transfection of 293 T cells and tittered on TZM-bl cells as previously described [34, 52–59]. Humanized mice were exposed to 9 × 10^4^ TCIU HIV-1_JR − CSF_ or HIV-1_CH040_ via tail vein injection (200 µL). HIV-RNA levels were measured in the peripheral blood plasma of mice by RT–qPCR using a TaqMan RNA to-CT 1-step kit (Applied Biosystems) at time of tissue harvest as previously described [34, 52–59]. Briefly, peripheral blood was collected into tubes containing 5 mM EDTA and plasma isolated following centrifugation (2,000 RPM, 5 min). The sequences of the forward and reverse primers and the TaqMan probe for PCR amplification and detection of HIV gag RNA were: 5′-CATGTTTTCAGCATTATCAGAAGGA-3′, 5′-TGCTTGATGTCCCCCCACT-3′, and 5′-FAM-CCACCCCACAAGATTTAAACACCAT-GCTAA-Q-3′, respectively.

### Tissue collection and processing for histological analysis

Humanized mice were euthanized 2 weeks (HIV-1_JR − CSF_, *n* = 4; HIV-1_CH040_, *n* = 4) or 8 weeks (HIV-1_JR − CSF_, *n* = 4; HIV-1_CH040_, *n* = 4) after HIV-1 virus exposure. Peripheral blood was collected prior to transcardial perfusion with 20–30 mL 1x phosphate buffer solution (PBS), followed by 20–30 mL ice cold 4% paraformaldehyde (in PBS). Brains were extracted, postfixed in 4% paraformaldehyde for 6 h at 4 °C, washed 3x with PBS for 1 h, and then incubated in 30% sucrose for 2 d. Subsequently, brains were hemisected in the midsagittal plane and the two hemispheres were embedded in Tissue-Tek optical cutting temperature (OCT) compound, frozen using dry ice, and stored at -80 °C until cut. Sagittal brain sections (30 μm) containing the frontal cortex, hippocampus, dorsal striatum, cerebellum, and spinal cord (portions of the C1–C5) were cut on a Leica CM3050S cryostat (Leica, Deerfield, IL). Sections for each subject were placed in a PBS filled 12-well plate, 5 sections per well, and sealed for storage at 4 °C until immunolabeling.

#### Human immune cell labeling and HIV p24 detection

Free-floating sections were first incubated for antigen retrieval with 1x DIVA Decloaker (DV2004, Biocare Medical) for 30 min at 95 °C and an additional 30 min at RT, washed 3x with ddH_2_O for 5 min, followed by exposure to blocking buffer for 30 min (Background Sniper, Biocare Medical). Sections were then incubated overnight at 4 °C with the following primary antibodies diluted in TNP blocking buffer containing 2% Sniper reagent: hCD3 (rabbit, Thermo Scientific, #RM9107S, 1:150) for the detection of human T cells, hCD68 (rabbit, Thermo Scientific, #MBS303274, 1:200) for the detection of human macrophages, p24 (mouse, Aligent Technologies, #M085701-1, 1:36) for the detection of HIV-infected cells. The primary antibodies were detected using secondary antibodies as follows: donkey-anti-rabbit Alexa 488 (ThermoFisher, #SA5-10038, green,1:200) and/or donkey-anti-mouse Alexa 650 (ThermoFisher, #SA5-10169, red, 1:200). Cell nuclei were visualized with Hoechst 33,342 (Molecular Probes, H3570, exposed for 3 min). Tissue sections were washed thoroughly with PBS, mounted on Superfrost Plus glass microscopic slides (Fisher Scientific, #12-550-15) and coverslipped with antifade mounting medium (VectaShield, #H-1400). Confocal immunofluorescent images z-stacks were acquired using a Zeiss LSM800 T-PMT laser scanning confocal microscope (Zeiss, Thornwood, NY) equipped with a 63x objective. Orthogonal projections were taken from each image stack using ZEN 2010 Blue Edition software (Zeiss, Thornwood, NY).

#### Multiplex fluorescent in situ hybridization assay

Multiplex fluorescent in situ hybridization was performed using an RNAscope™ Multiplex Fluorescent V2 Assay (ACDBio) according to the manufacturers’ protocol. In brief, fixed frozen sagittal brain sections were prepared at 12 μm width, mounted on Superfrost Plus glass microscopic slides (Fisher Scientific), and kept at -20 °C. Slides were post-fixed and dehydrated in increasing concentrations of ethanol. After dehydration, brain slices were treated with hydrogen peroxide before immersion with target retrieval reagent (ACDBio) for 5 min at 97–99 °C. Afterwards, slides were treated with protease III reagent (ACDBio) for 30 min at 40 °C. Following tissue preparation and pretreatment, slides were incubated with RNA transcript probes targeting HIV Gag for the detection of HIV-infected cells, human CD68 for the detection of human macrophages, and human PTPRC (CD45) for the detection of human hematopoietic cells. Slides were incubated overnight (~ 18 h) in saline sodium citrate buffer. Amplification of the probe signals was performed by addition of Amp1, Amp2, and Amp3 reagents (ACDBio) at 40 °C. Each probe then underwent (1) development with the appropriate HRP, (2) labeling with a TSA fluorophore dye and (3) exposure to an HRP blocker reagent. The CD68 probe was labeled with TSA 570 (ACDBio, 1:1500), the HIV Gag probe with TSA 520 (ACDBio, 1:6000), and the PTPRC/CD45 probe with TSA 650 (ACDBio, 1:1500). Tissue sections were counterstained with DAPI (ACDBio) then mounted with ProLong Gold Antifade Mountant (Invitrogen) before the placing of coverslips. Images were collected with a Zeiss LSM800 laser scanning confocal microscope using a 20x objective lens and then processed using ImageJ/FIJI software (v.2.14.0) [60]. A negative control probe set consisting of targets for the dihydrodipicolinate reductase (dapB) gene of the soil bacteria Bacillus subtilis was utilized to ensure signal specificity.

#### Murine cell labeling of neurons, astrocytes, and microglia

Free-floating sections were first incubated in 0.5% H_2_O_2_ for 30 min, in 1% H_2_O_2_ for 60 min, and again in 0.5% H_2_O_2_ for 30 min, washed 3x with PBS (1x) for 5 min, followed by exposure to blocking buffer for 1 h (PBS with 3% normal goat serum and 0.5% Triton X-100). Sections were then incubated overnight at 4 °C with the following primary antibodies diluted in blocking buffer containing normal goat serum: ionized calcium-binding adapter molecule 1 (Iba-1, rabbit, Wako, #019-19741, 1:500) for the detection of microglia, glia fibrillary acidic protein (GFAP, rabbit, Millipore, #AB5804, 1:500) for the detection of astrocytes, neuronal nuclear (NeuN, mouse, Millipore, #MAB377, 1:500) for the detection of neurons, or microtubule-associated protein 2, ab (MAP2ab, mouse, Millipore, #MAB378, 1:500) for the detection of neuronal dendrites. The primary antibodies were detected using secondary antibodies as follows: goat-anti-rabbit Alexa 594 (ThermoFisher, #A11012, red,1:500) and/or goat-anti-mouse Alexa 488 (ThermoFisher, #A21121, green, 1:500). The secondary antibodies were diluted in goat blocking buffer and applied to the sections for 1 h at RT. Cell nuclei were visualized with Hoechst 33,342 (Molecular Probes, H3570, exposed for 3 min). Tissue sections were washed thoroughly with PBS, mounted on Superfrost Plus glass microscopic slides (Fisher Scientific) and coverslipped with antifade mounting medium (VectaShield, #H-1400). Confocal immunofluorescent images were acquired using a Zeiss LSM800 T-PMT laser scanning confocal microscope (Zeiss, Thornwood, NY) equipped with a 20x objective. Images were acquired by using identical parameters for all groups (i.e., identical objective, zoom, laser intensity, gain, offset, and scan speed) optimized for control tissues. ZEN 2010 blue edition software (Zeiss, Thornwood, NY) was used to collect the images. For all five CNS regions, one image was sampled per section from 5 to 8 sagittal sections, spaced 300 μm apart, per animal.

Iba-1^+^ microglial cell bodies and/or NeuN^+^ neuronal cell bodies containing Hoechst-stained nuclei were counted by two experimenters blinded to treatment groups. Reliability (Cronbach’s α) of Iba-1^+^ microglial cell counts and NeuN^+^ neuronal cell counts between the two experimenters was assessed for all CNS regions ranging between 0.816 and to 0.969 (Iba-1^+^ microglia: frontal cortex: α = 0.950, hippocampus: α = 0.892, striatum: α = 0.909, cerebellum: α = 0.816, spinal cord: α = 0.900; NeuN^+^ neurons: frontal cortex: α = 0.956, hippocampus: α = 0.854, striatum: α = 0.969, cerebellum: α = 0.953, spinal cord: α = 0.937). Data presented as the number of Iba-1^+^ microglia or number of NeuN^+^ neurons represent the average counts from both experimenters.

For MAP2ab and GFAP the entire image was used as region of interest and processed using ImageJ [61] to quantify the density of staining per pixel in each image. Mean fluorescent intensities (MFI) were determined with ImageJ without digital manipulation. Data represent the mean fluorescent intensities for MAP2ab and GFAP.

### Statistical analysis

All data are presented as mean ± the standard error of the mean (SEM). Datasets were analyzed by one-way analysis of variances (ANOVAs) with treatment group (5 levels: No virus infection, 2 weeks HIV-1_CH040_ infection, 8 weeks HIV-1_CH040_ infection, 2 weeks HIV-1_JR − CSF_ infection, 8 weeks HIV-1_JR − CSF_ infection) as a between-subjects factor, except for plasma viral load. For plasma viral load a one-way ANOVA was conducted for the infected groups only (4 levels: 2 weeks HIV-1_CH040_ infection, 8 weeks HIV-1_CH040_ infection, 2 weeks HIV-1_JR − CSF_ infection, 8 weeks HIV-1_JR − CSF_ infection) to assess differences in HIV infection by both viral strains. ANOVAs were followed by Bonferroni’s post hoc tests for group comparisons when appropriate. The relationship between plasma viral load and the four CNS neuronal and glial cell markers across CNS regions as well as the association between the various CNS markers within each CNS region were assessed via Pearson correlation analyses. An alpha level of *p* ≤ 0.05 was considered significant for all statistical tests. SPSS Statistics 25 (IBM, Chicago, IL) and Prism GraphPad 8.0 (San Diego, CA) were used for data analysis and data graphing, respectively.

## Results

### Systemic infection of humanized mice by HIV

CNS pathology was evaluated in BLT humanized mice infected with HIV-1_JR-CSF_, an early passage CCR-5 tropic strain isolated from CSF, or HIV-1_CH040_, a CCR-5 tropic transmitted/founder virus strain. Humanized mice were prepared as previously reported [34, 52–59]. The levels of human hematopoietic cells (hCD45^+^), human T cells (hCD3^+^), and human CD4^+^ T cells (hCD3^+^ and hCD4^+^) in peripheral blood were determined prior to HIV exposure (Table 1). One-way ANOVAs revealed no significant differences between the different mouse groups, confirming that the frequency of human hematopoietic and T cells in peripheral blood were similar across all five groups.

**Table 1:** Humanized mouse peripheral blood humanization levels and HIV RNA levels in the plasma prior infection

Mouse	Sex	Virus(es)	Time Point		*HIV-RNA copies/mL		%hCD45^+^		%hCD3^+^ of hCD45^+^		%hCD4^+^ of hCD3^+^/hCD45^+^
Naive 1	F	No Virus	Naive		N/A		80.1		44.8		86.0
Naive 2	F	No Virus	Naive		N/A		83.6		60.7		84.8
Naive 3	M	No Virus	Naive		N/A		55.7		89.5		79.8
Naive 4	M	No Virus	Naive		N/A		70.0		79.7		82.9
Mean ± SEM					N/A		68.7 ± 10.0		83.4 ± 1.4		72.4 ± 6.3
CH040 1	F	CH040	2		33,559,417		71.3		84.4		86.9
CH040 2	F	CH040	2		1,241,086		39.6		99.9		78.0
CH040 3	M	CH040	2		17,913,052		58.2		90.7		80.7
CH040 4	F	CH040	2		7,780,547		70.1		76.3		83.1
Mean ± SEM					15,123,525.5 ± 7,037,429.8		87.8 ± 5.0		82.2 ± 1.9		59.8 ± 7.4
CH040 1	F	CH040	8		1,889,667		82.1		56.3		75.2
CH040 2	F	CH040	8		945,117		76.5		66.2		77.6
CH040 3	M	CH040	8		5,712,490		61.0		59.6		78.4
CH040 4	F	CH040	8		926,170		83.3		71.8		79.2
Mean ± SEM					2,368,361.0 ± 1,137,170.7		63.5 ± 3.5		77.6 ± 0.9		75.7 ± 5.1
Mean ± SEM for all HIV-1_CH040_ infected mice					8,745,943.3 ± 4,086,588.1		75.7 ± 5.4		79.9 ± 1.3		67.7 ± 5.2
JR-CSF 1	M	JR-CSF	2		5,528,643		40.7		92.2		79.4
JR-CSF 2	F	JR-CSF	2		2,272,120		56.2		67.5		78.0
JR-CSF 3	F	JR-CSF	2		4,714,425		66.6		48.7		86.8
JR-CSF 4	F	JR-CSF	2		2,710,522		72.3		57.5		87.3
Mean ± SEM					3,806,427.5 ± 782,389.2		66.5 ± 9.4		82.9 ± 2.4		59.0 ± 6.9
JR-CSF 1	F	JR-CSF	8		1,073,121		74.9		62.8		80.7
JR-CSF 2	M	JR-CSF	8		1,019,760		52.9		93.7		76.9
JR-CSF 3	F	JR-CSF	8		411,746		78.0		74.5		80.6
JR-CSF 4	F	JR-CSF	8		807,367		81.4		73.9		80.8
Mean ± SEM					827,998.5 ± 150,152.5		76.2 ± 6.4		79.8 ± 1.0		71.8 ± 6.4
Mean ± SEM for all HIV-1_JR − CSF_ infected mice					2,317,213.0 ± 672,923.0		71.4 ± 5.6		81.3 ± 1.3		65.4 ± 5.0

*HIV-RNA copies/mL taken at time of harvest 2- or 8-weeks post-infection. N/A, not applicable; F, female; M, male

Humanized mice were infected intravenously with HIV-1_JR-CSF_ or HIV-1_CH040_ and CNS pathology was analyzed in the brain and spinal cord at 2- or 8-weeks post-infection. Both virus strains established efficient and robust infection in humanized mice as determined by their viral load in peripheral blood (Table 1). Further, a one-way ANOVA demonstrated a trend towards significance, *F*(1, 3) = 3.3, *p* = 0.058, with viral load levels in HIV-1_CH040_ infected mice being higher compared to HIV-1_JR-CSF_ infected mice at 2-weeks and 8-weeks post-infection (Table 1). Bonferroni’s post hoc tests revealed no significant differences between groups.

### Detection of human immune cells and HIV in the brains of BLT mice

**Fig. 1:**
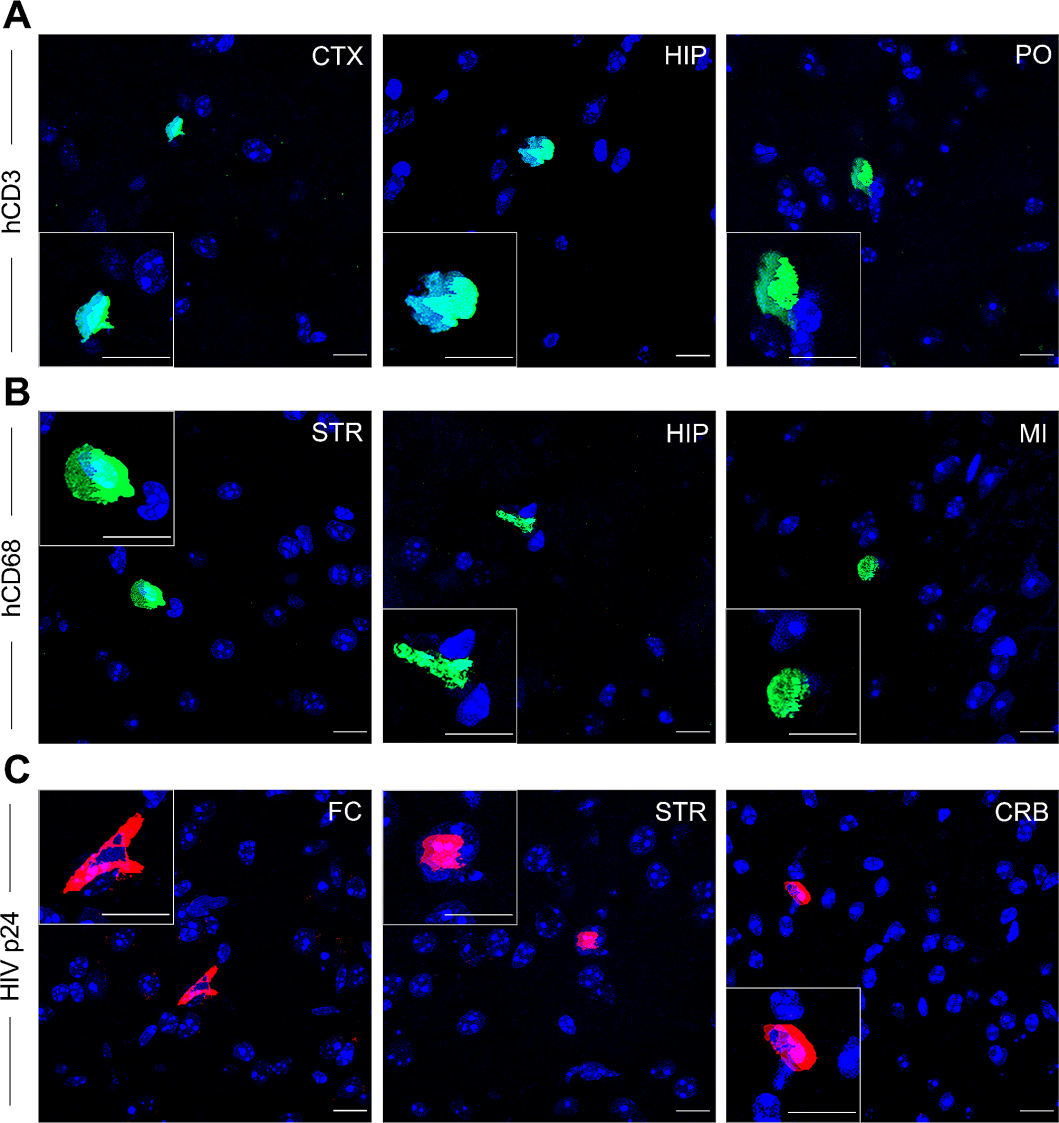
Human immune cells are distributed throughout the brains of BLT mice with HIV being present in the brain of infected BLT mice. Immunohistochemical analysis from humanized BLT mice demonstrate the presence of human T cells (hCD3^+^, green, **A**) and human macrophages (hCD68^+^, green, **B**) in various brain regions. Further brain sections reveal the presence of HIV p24^+^ cells (red, **C**) in various brain regions of BLT humanized mice infected with HIV-1_CH040_ at 2-weeks post-infection. Scale bars: 10 μm. Original magnification at 63x, x2 (insets). FC, frontal cortex; CTX, cortex; HIP, hippocampus; STR, striatum; CRB, cerebellum; MI, midbrain; PO, pons

**Fig. 2:**
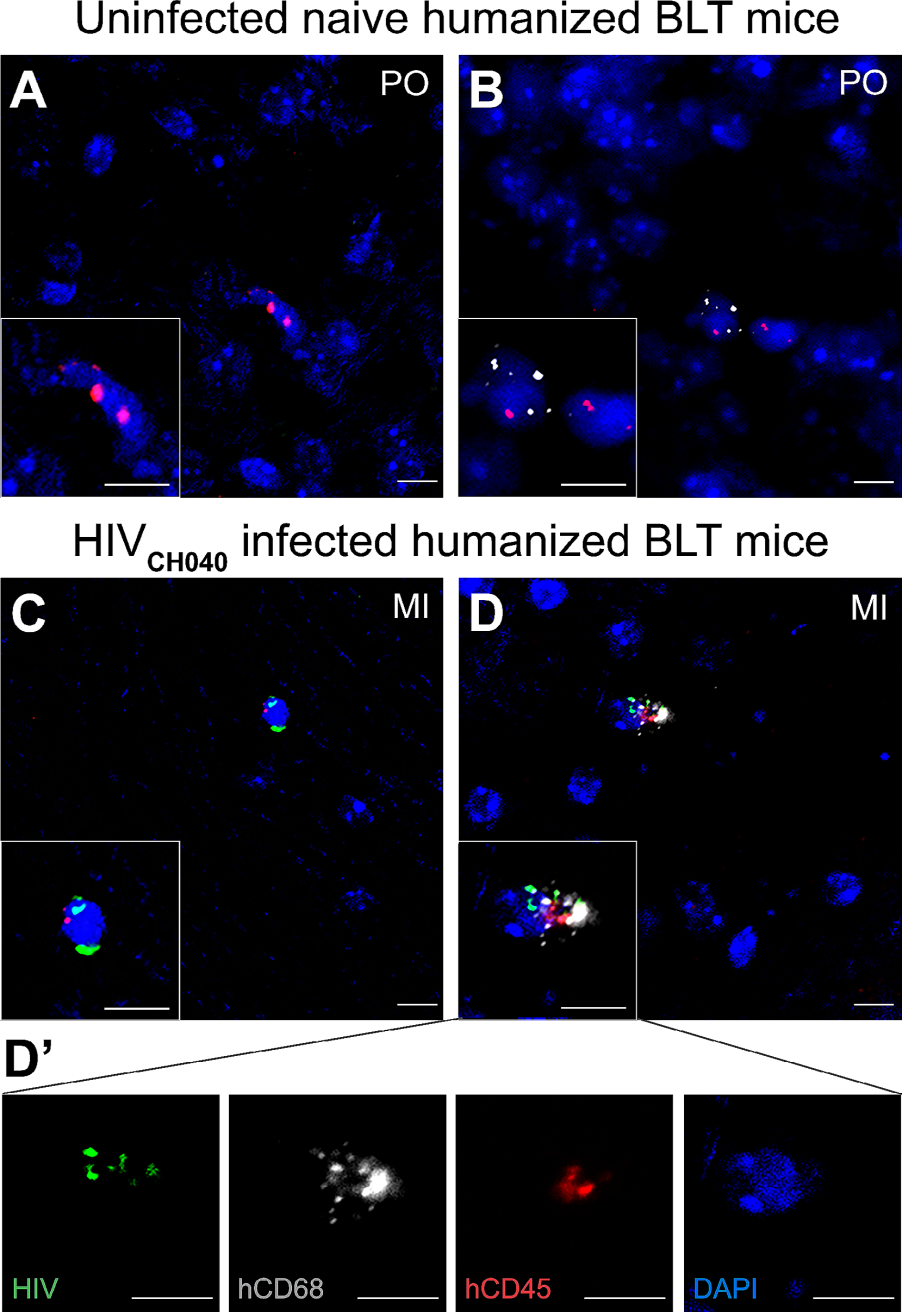
HIV infected human hCD68^+^/hCD45^+^ macrophages are present in the brain of HIV-1_CH040_ infected BLT mice. RNAScope analysis for hCD45 (red), hCD68 (gray), and HIV (green) expression demonstrate the presence of human hCD45^+^ immune cells (**A**) and human hCD68^+^/hCD45^+^ macrophages (**B**) in the pons of uninfected mice. No HIV RNA was observed in brain cells from uninfected mice. Analysis of humanized BLT mice infected with HIV-1_CH040_ demonstrate the presence of HIV-infected hCD45^+^ cells (**C**) and HIV-infected hCD68^+^/hCD45^+^ cells (**D, D’**) in the midbrain. Scale bars: 10 μm. Original magnification at 20x zoomed in, x2 (insets). MI, midbrain; PO, pons

Immunohistochemistry (Fig. 1) and RNAScope (Fig. 2) were conducted to demonstrate the presence of human immune cells and HIV infection in the brain of BLT mice. Results from immunohistochemistry demonstrate the presence of human hCD3^+^ T cells (Fig. 1A) and human hCD68^+^ macrophages (Fig. 1B) in various brain regions, including the cortex, hippocampus, striatum, midbrain, and pons, indicating that the brains of the humanized mice were repopulated with human immune cells as previously described [34]. Additionally, HIV-infected cells were also observed in multiple brain regions (Fig. 1C), demonstrating HIV neuroinvasion in the infected BLT mice. When conducting RNAScope we observed infection of human cells (hCD45^+^) in the brain of HIV-infected humanized mice (Fig. 2C). Importantly, we also noted the presence of HIV-infected hCD68^+^/hCD45^+^ macrophages in the brain of HIV-1_CH040_ infected humanized mice (Fig. 2D, D’). In contrast, while hCD45^+^(Fig. 2A) and hCD68^+^/hCD45^+^ (Fig. 2B) cells were observed in the brain of uninfected humanized mice, no HIV-infected cells were detected.

### Neuronal loss and injury in the CNS of HIV-infected humanized mice

**Fig. 3:**
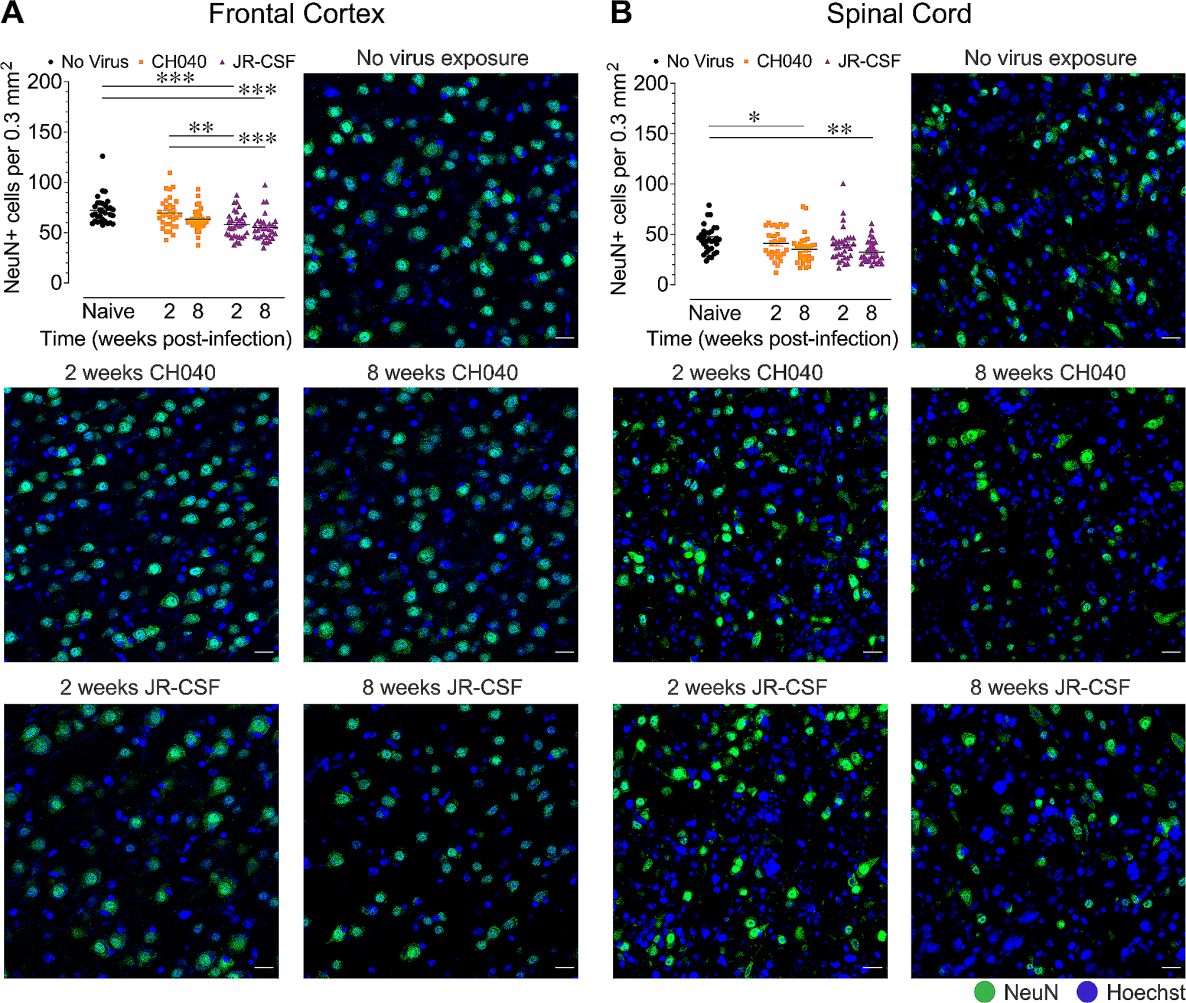
HIV infection induces neuronal loss in the frontal cortex and spinal cord. Quantification of NeuN^+^ neurons with representative images of NeuN^+^ neurons (green, Hoechst/cell nuclei in blue) for all five BLT humanized mouse groups in the frontal cortex **(A)** and spinal cord **(B)**. Statistical significance was assessed by one-way ANOVAs followed by Bonferroni’s post hoc tests when appropriate; *p < 0.05, **p < 0.01, ***p < 0.001. Sample derived from 5–8 sections per mouse with *n* = 4 mice per group. NeuN: neuronal nuclear protein. Scale bars = 20 μm

**Fig. 4:**
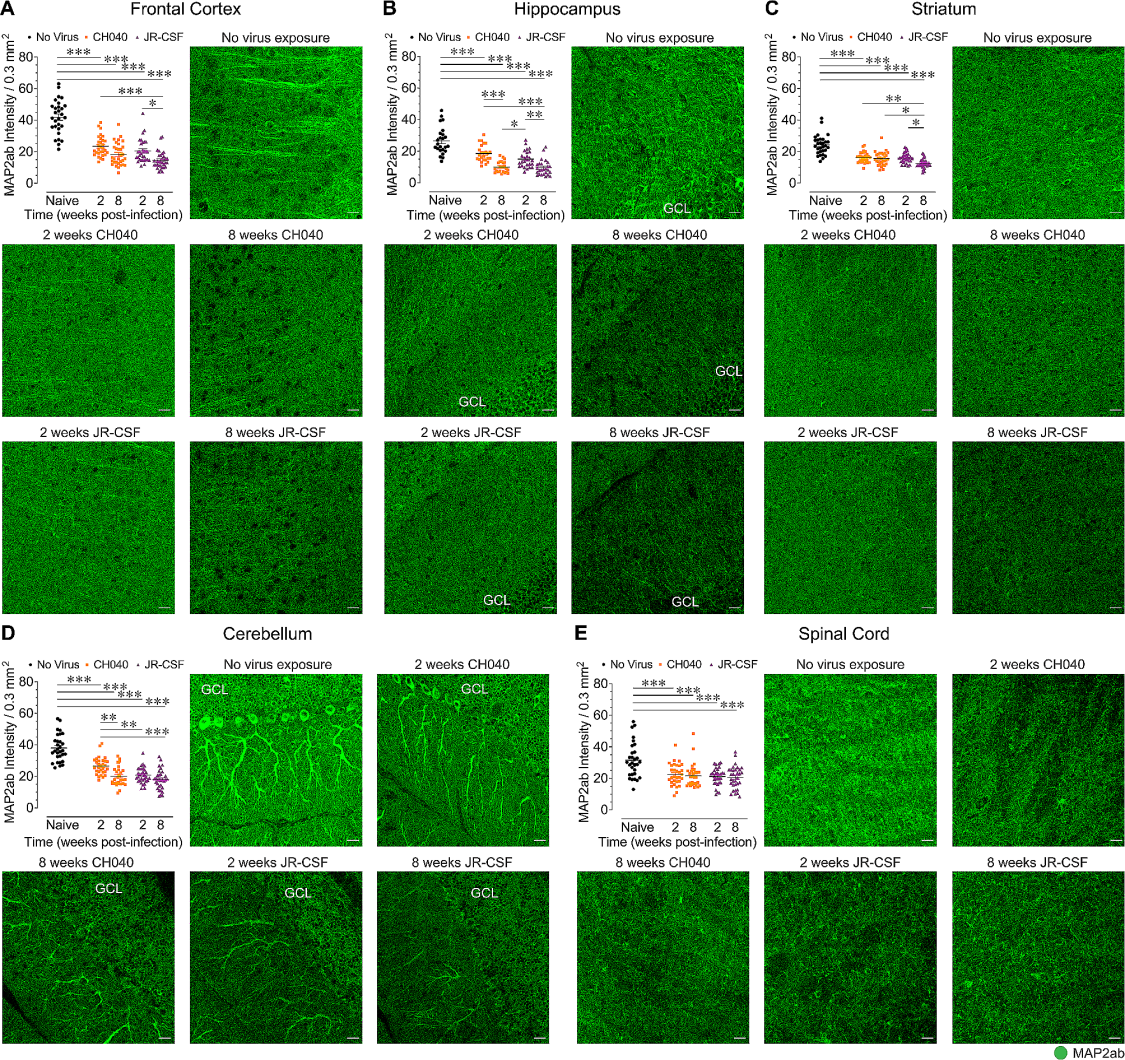
HIV-1_CH040_ and HIV-1_JR − CSF_ viruses induce neuronal dendritic injury in all CNS regions which increases with the duration of infection. Quantification of mean fluorescence intensity for neuronal dendritic MAP2ab (arbitrary units) with representative images of neuronal dendritic MAP2ab expression levels for all five BLT humanized mouse groups in the frontal cortex **(A)**, hippocampus **(B)**, striatum **(C)**, cerebellum **(D)**, and spinal cord **(E)**. Statistical significance was assessed by one-way ANOVAs followed by Bonferroni’s post hoc tests when appropriate; *p < 0.05, **p < 0.01, ***p < 0.001. Sample derived from 5–8 sections per mouse with *n* = 4 mice per group. MAP2ab: microtubule-associated protein 2ab. Scale bars = 20 μm

To assess neuronal integrity in brain regions and the spinal cord (*n* = 4 per group/5–8 sections each) of HIV-infected humanized mice, immunofluorescence was conducted to quantify the number of NeuN^+^ immunoreactive neurons (Fig. 3) and the mean fluorescence intensity of MAP2ab^+^ immunoreactive neuronal dendrites (Fig. 4) in naive control and HIV-infected mice.

*HIV infection results in a reduction in NeuN*^*+*^*neurons*. Significant differences between groups were noted for the number of NeuN^+^ neurons in the frontal cortex, *F*(4, 153) = 9.5, *p* < 0.001, and spinal cord, *F*(4, 154) = 4.1, *p* = 0.004 (Fig. 3). The frontal cortex was specifically affected in HIV-1_JR − CSF_ infected humanized mice, which had significantly lower numbers of NeuN^+^ neurons compared to naive mice, at 2-weeks (*p* < 0.001) and 8-weeks (*p* < 0.001) post-exposure and compared to HIV-1_CH040_ infected mice at 2-weeks post-exposure (*p* = 0.007 and *p* < 0.001, respectively). For the spinal cord, the timing of post-infection was critical to induce neuronal loss, with lower numbers of NeuN^+^ neurons only being observed at 8-weeks post-infection in HIV-1_CH040_ (*p* = 0.052) and HIV-1_JR − CSF_ (*p* = 0.003) infected mice compared to naive mice. No significant effects on neuronal number were noted for the hippocampus, striatum, or cerebellum (Supplemental Figure S1). Thus, loss of neurons during HIV infection was limited to the frontal cortex and spinal cord, with HIV-1_JR − CSF_ specifically affecting the frontal cortex and both HIV-1_JR − CSF_ and HIV-1_CH040_ causing neuronal loss in the spinal cord after 8 weeks of infection.

*HIV infection results in neuronal dendritic injury*. Significant differences were noted in the levels of MAP2ab expression in all CNS regions between infected and naive mice [frontal cortex, *F*(4, 148) = 59.48, *p* < 0.001; hippocampus, *F*(4, 119) = 36.55, *p* < 0.001; striatum, *F*(4, 150) = 34.45, *p* < 0.001; cerebellum, *F*(4, 148) = 49.49, *p* < 0.001; spinal cord, *F*(4, 150) = 10.30, *p* < 0.001; Fig. 4]. Group comparisons for all CNS regions demonstrated that naive humanized mice displayed significantly higher MAP2ab expression levels compared to HIV-1_JR − CSF_ and HIV-1_CH040_ infected mice analyzed at 2-weeks and 8-weeks post-infection (*p*’s < 0.001), suggesting that neuronal dendritic injury was induced by both virus strains and present at both time points of analysis. Interestingly, the most pronounced damage to neuronal dendrites across all brain regions was observed in HIV-1_JR − CSF_ infected mice analyzed at 8-weeks post-infection. For the frontal cortex, hippocampus, and striatum, HIV-1_JR − CSF_ infected mice analyzed at 8-weeks post-infection showed lower MAP2ab fluorescence levels compared to HIV-1_JR − CSF_ infected mice analyzed at 2-weeks post-infection (frontal cortex, *p* = 0.021; hippocampus, *p* = 0.007; striatum, *p* = 0.014). Additionally, at 8-weeks post-infection, HIV-1_JR − CSF_ infected mice showed lower MAP2ab expression levels in the striatum compared to HIV-1_CH040_ infected mice (*p* = 0.037). Lastly, lower MAP2ab immunoreactivity was also noted for HIV-1_CH040_ infected mice analyzed at 8-weeks post-infection in the hippocampus (*p* < 0.001) and cerebellum (*p* = 0.028) compared to HIV-1_CH040_ infected mice analyzed at 2-weeks post-infection. Thus, neuronal dendritic injury was induced by both viral strains at both time points in all CNS regions. However, after 8-weeks post-infection dendritic injury was most prominent in mice infected with HIV-1_JR − CSF_.

Overall, our results show that whereas HIV-induced neuronal loss was noted only in the frontal cortex and spinal cord, infection with HIV-1_JR − CSF_ and HIV-1_CH040_ induced neuronal dendritic injury in all CNS regions, which increased with the duration of infection. Further, infection with HIV-1_JR − CSF_ appeared to have the most deleterious effects on neuronal health in the CNS regions assessed, specifically in animals analyzed at 8-weeks post-infection. These results suggest that viral strains have differential pathological effects on neuronal health.

### HIV infection results in neuroinflammation in the CNS

**Fig. 5:**
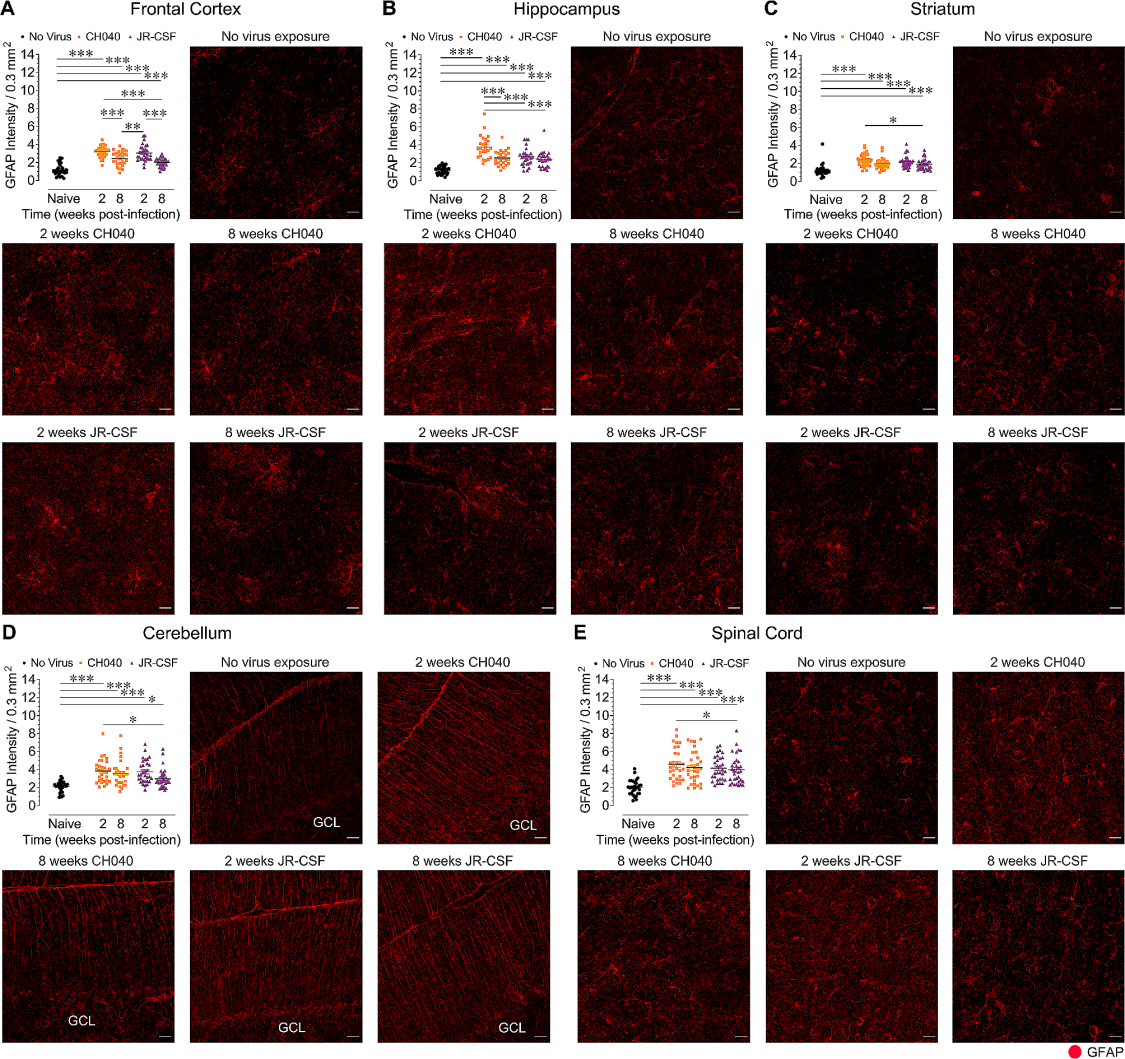
Infection with HIV-1_CH040_ and HIV-1_JR − CSF_ induces astrocytosis in all CNS regions. Quantification of mean fluorescence intensity for astrocytic GFAP (arbitrary units) with representative images of astrocytic GFAP expression levels for all five BLT humanized mouse groups in the frontal cortex **(A)**, hippocampus **(B)**, striatum **(C)**, cerebellum **(D)**, and spinal cord **(E)**. Statistical significance was assessed by one-way ANOVAs followed by Bonferroni’s post hoc tests when appropriate; *p < 0.05, **p < 0.01, ***p < 0.001. Sample derived from 5–8 sections per mouse with *n* = 4 mice per group. GFAP: glial fibrillary acidic protein. Scale bars = 20 μm

**Fig. 6:**
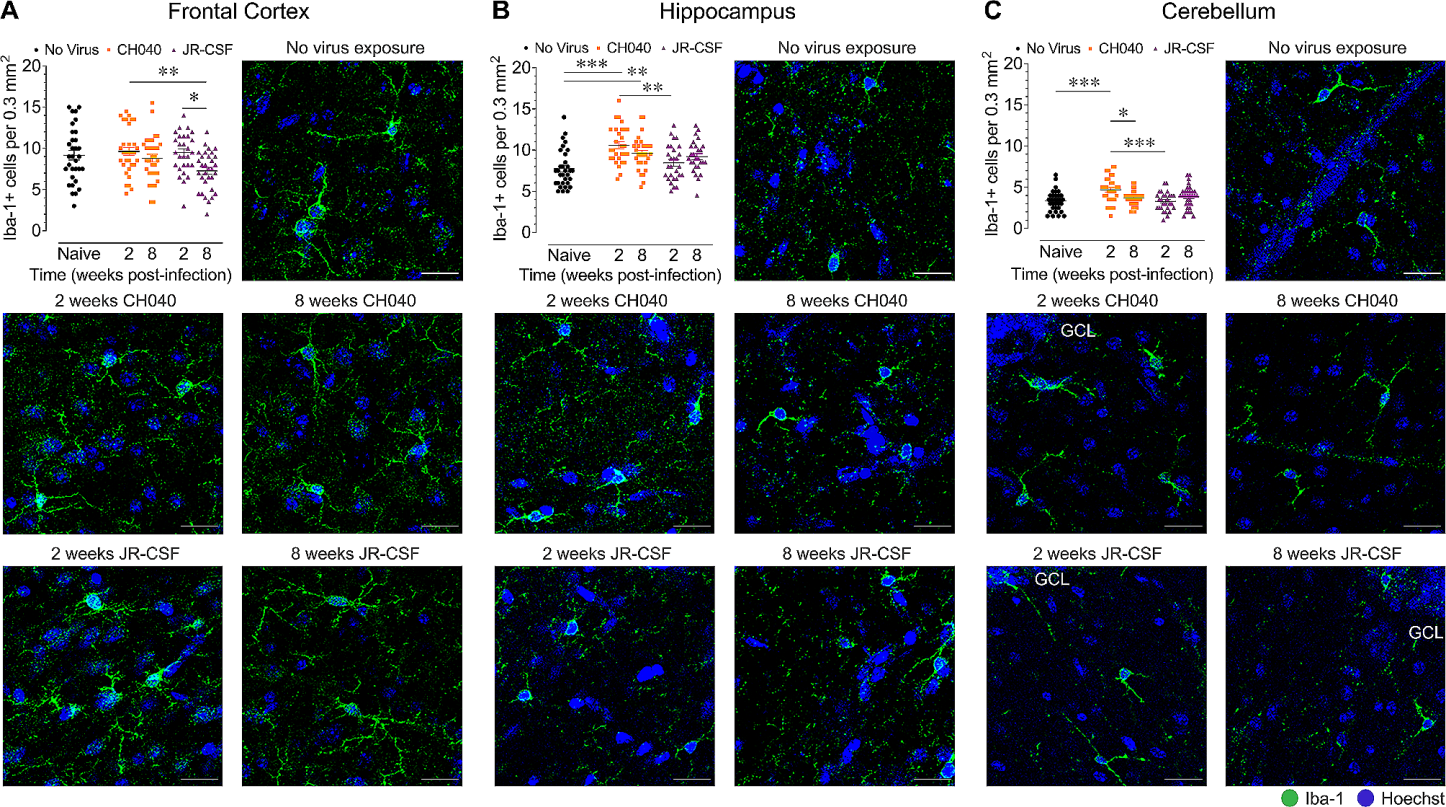
Microglial cell activation is CNS region specific and more prominent in HIV-1_CH040_ infected mice. Quantification of Iba-1^+^ microglia with representative images at higher magnification of Iba-1^+^ microglia (green, Hoechst/cell nuclei in blue) for all five BLT humanized mouse groups in the frontal cortex **(A)**, hippocampus **(B)**, and cerebellum **(C)**. Statistical significance was assessed by one-way ANOVAs followed by Bonferroni’s post hoc tests when appropriate; *p < 0.05, **p < 0.01, ***p < 0.001. Sample derived from 5–8 sections per mouse with *n* = 4 mice per group. Iba-1: ionized calcium binding adaptor molecule 1. Scale bars = 20 μm

We next assessed neuroinflammatory responses in brain regions and the spinal cord (*n* = 4 per group/5–8 sections each) by quantifying the mean fluorescence intensity of astrocytic GFAP^+^ levels in mouse cells (Fig. 5) and the number of mouse Iba-1^+^ microglia (Fig. 6).

*HIV infection induces astrogliosis.* Significant differences between groups were noted for GFAP levels in all CNS regions analyzed [frontal cortex, *F*(4, 150) = 44.28, *p* < 0.001; hippocampus, *F*(4, 125) = 26.16, *p* < 0.001; striatum, *F*(4, 150) = 14.45, *p* < 0.001; cerebellum, *F*(4, 148) = 11.63, *p* < 0.001; spinal cord, *F*(4, 150) = 14.37, *p* < 0.001; Fig. 5]. Group comparisons for all CNS regions demonstrated that naive control mice displayed significantly lower GFAP fluorescence levels compared to HIV-1_JR − CSF_ and HIV-1_CH040_ infected mice (*p*’s < 0.05), suggesting that astrogliosis was induced by both virus strains and was present at both time points, 2-weeks and 8-weeks post-infection. Interestingly, astrocytic GFAP astrogliosis was most pronounced in the frontal cortex and hippocampus in HIV-infected mice analyzed at 2-weeks post-infection. Specifically, at 2-weeks post-infection HIV-1_CH040_ infected mice showed higher GFAP fluorescence in the frontal cortex and hippocampus compared to HIV-1_CH040_ infected mice analyzed at 8-weeks post-infection (*p*’s < 0.001). Additionally, at 2-weeks post-infection higher GFAP^+^ expression levels were also noted for HIV-1_JR − CSF_ infected mice in the frontal cortex compared to HIV-1_JR − CSF_ infected mice analyzed at 8-weeks post-infection (*p* < 0.001) . Thus, astrogliosis was induced in all CNS regions by both viral strains at both time points, with the most prominent effects being observed at 2-weeks post-infection.

*HIV infection results in microglia activation.* While no significant differences in the number of Iba-1^+^ microglia were observed between naive control mice and HIV-infected mice in the frontal cortex, striatum, or spinal cord, the number of Iba-1^+^ microglia were significantly higher in HIV-1_CH040_ infected mice in the hippocampus and cerebellum (Fig. 6, Supplementary Figure S2 and S3). Specifically, in the hippocampus, infection with HIV-1_CH040_ resulted in higher number of Iba-1^+^ microglia at both time points compared to naive control humanized mice (2 wks CH040, *p* < 0.001; 8 wks CH040, *p* = 0.009) (Fig. 6). In the cerebellum, higher number of Iba-1^+^ microglia were observed in HIV-1_CH040_ infected mice analyzed at 2-weeks post-infection compared to naive control mice (*p* < 0.001) (Fig. 6).

In summary, both viral strains induced astrogliosis in all CNS regions, with the most prominent effects being seen in HIV-1_CH040_ infected mice at 2-weeks post-infection. Upregulation of Iba-1^+^ microglia were CNS region specific and again more so noted in HIV-1_CH040_ infected mice and at 2-weeks post-infection. These results suggest that some virus strains may have more neuroinflammatory effects especially during acute infection.

### Plasma viral loads are not indicative of HIV-1-induced CNS pathology

As plasma viral load levels trended higher in HIV-1_CH040_ infected mice compared to HIV-1_JR − CSF_ infected mice at both time points (Table 1, one-way ANOVA; *p* = 0.058), we assessed the relationship between levels of plasma viral load, humanized engraftment levels, and CNS pathology. Pearson correlations were conducted for the four infected humanized mouse groups between plasma viral load, levels of human hematopoietic and T cells in peripheral blood (%hCD45^+^, %hCD3^+^ of hCD45^+^, %hCD4^+^ of hCD3^+^/hCD45^+^), and each of the four CNS markers (MAP2ab, NeuN, GFAP, Iba-1) across all five CNS regions. No significant relationship was noted between plasma viral load, human hematopoietic and/or T cell levels, and any of the CNS markers.

To assess the relationship between neuroinflammation and neuronal health, Pearson correlations were conducted for the four CNS markers across all five humanized mouse groups, separately for each CNS region. Only one positive correlation was detected between neuroinflammation and neuronal health in the frontal cortex, with a higher number of microglial cells being associated with higher numbers of NeuN^+^ neurons (frontal cortex, Iba-1 vs. NeuN, *r* = 0.447, *p* = 0.048). Furthermore, within each of the CNS markers for neuronal health or neuroinflammation, positive correlations were demonstrated in selected CNS regions as follows; high MAP2ab expression levels was associated with high numbers of NeuN^+^ cells in the frontal cortex (MAP2ab vs. NeuN, *r* = 0.581, *p* = 0.007) and the spinal cord (MAP2ab vs. NeuN, *r* = 0.462, *p* = 0.040), and high GFAP signal was associated with high number of Iba-1^+^ microglia in the hippocampus (GFAP vs. Iba-1, *r* = 0.563, *p* = 0.010).

Overall, no association was noted between plasma viral load and CNS pathology in any of the five CNS regions, suggesting that the levels of viral load found in plasma are not indicative of HIV-1-induced CNS pathology. Further, neuroinflammatory effects appear to have a weak relationship with neuronal health, but neuronal markers, MAP2ab levels and NeuN^+^ neurons, or neuroinflammation markers, GFAP levels and Iba-1^+^ microglia, demonstrate some positive association for selected CNS regions.

## Discussion

The present study demonstrates that HIV-1_JR − CSF_ and HIV-1_CH040_ infection in BLT humanized mice induces neuronal injury and neuroinflammation. We observed infected HIV p24^+^ cells in various brain regions, including the frontal cortex, striatum, and cerebellum, of HIV-infected humanized mice. Co-staining for HIV and the macrophage marker hCD68 demonstrated the presence of HIV^+^ human macrophages in the brain of HIV-1_CH040_ infected humanized mice. Whereas the current study did not assess HIV viral load in the CNS, past publications quantified HIV RNA and DNA levels in the whole brain of BLT humanized mice infected with HIV-1_CH040_ or HIV-1_JR − CSF_ and observed a positive association between plasma viral load and the levels of cell-associated HIV RNA in the brain in the absence of ART [34]. Interestingly, the present study did not find a significant relationship between HIV-induced CNS pathologies and plasma viral load or the levels of human hematopoietic and/or T cell levels in peripheral blood. The lack of a correlation between viral load and neurodegeneration has also been reported in the humanized NSG mouse model [48]. Indeed, multiple studies, including PLWH and non-human primate simian immunodeficiency virus studies, have reported that instead of plasma viral load, CSF viral RNA is associated with the development of neuropathology or cognitive deficits [62–64]. Thus, as plasma viral load is not always associated with HIV RNA in the brain [48], especially under cART [34], future studies should aim to correlate HIV DNA or RNA levels in the brain with induced effects on CNS pathology.

We show that both HIV-1 strains induced neuronal dendritic injury across all CNS regions following HIV infection. However, decreased MAP2ab signal was specifically noted at 8-weeks post-infection, and particularly for mice infected with HIV-1_JR − CSF_, as demonstrated for the frontal cortex and striatum at 8-weeks post-infection (Fig. 4A, C). The finding that infection with HIV-1_JR − CSF_ induces more deficits on neuronal health for specific CNS regions is also supported by NeuN^+^ neuron counts. Whereas only the frontal cortex and spinal cord demonstrated significant neuronal loss, the effect in the frontal cortex was specific to mice infected with HIV-1_JR − CSF_, showing lower number of NeuN^+^ neurons compared to the naive humanized mouse group and the HIV-1_CH040_ infected group analyzed at 2-weeks post-infection (Fig. 3A). Interestingly, in the frontal cortex and spinal cord high MAP2ab signal also correlated with high number of NeuN^+^ neurons, as indicated demonstrating by a positive relationship of both CNS markers in these two CNS regions. This finding is interesting as cortical regions were previously suggested to be the primary site of neuronal damage due to HIV-1 infection [48]. Further, the deficits induced on neuronal health specifically in mice infected with HIV-1_JR − CSF_ suggest that HIV-1_JR − CSF_ infection has the potential of causing long-term CNS damage.

Compared to HIV-1_JR − CSF_, HIV-1_CH040_ infection induced more prominent neuroinflammation, including astrogliosis and microgliosis. Whereas infection with both viral strains induced astrogliosis in all CNS regions at both time points, HIV-1_CH040_ infected mice at 2-weeks post-infection showed the highest GFAP^+^ fluorescence signal consistently across CNS regions. Specifically, in the hippocampus, GFAP^+^ levels were significantly higher in HIV-1_CH040_ infected mice at 2-weeks post-exposure compared to all other groups (Fig. 5B). Further, upregulation of Iba-1^+^ microglia was noted in the hippocampus and cerebellum of HIV-1_CH040_ infected mice when compared to naive mice (Fig. 6B, C). The number of Iba-1^+^ microglia present in the five CNS regions of HIV-1_JR − CSF_ infected mice and naive mice did not significantly differ. Noteworthy, the Iba-1 antibody used in the current study shows cross-reactivity with human and mouse cells, and thus can stain human macrophages in the brain as positive for Iba-1. However, as these mice do not have human microglia [65, 66], the number of cells that are human compared to those that are mouse is orders of magnitude smaller. The neuroinflammatory effects, which were predominantly noted in animals infected with HIV-1_CH040_, may be attributed to the dual infection of CD4^+^ T cells and macrophages [33, 67–69], which in the brain can more readily increase neuroinflammatory responses [70, 71]. We previously demonstrated that HIV-1_CH040_ can infect human macrophages in vivo [33, 69]. Further, the noted neuroinflammatory effects that were most prominently found after 2-weeks, but not 8-weeks, post-infection, are potentially due to an innate immune tolerance in which the immune responsiveness in the CNS becomes increasingly tolerant to sustained HIV exposure [72]. This is supported by a previous study that demonstrated innate immune tolerance in 3 months exposed HIV-1 transactivator of transcription (Tat) transgenic mice, which was reflected in reductions in IL-1α, IL-12p40 and microglial reactivity compared to acute Tat exposure [72]. It should also be noted that the present study did not find an overall association between neuroinflammation and neuronal injury and demonstrated only a significant correlation in the frontal cortex when assessing associations separately for CNS regions, with higher number of microglial cells being associated with higher numbers of NeuN^+^ neurons. This is an unexpected finding, as high microglia activity has usually been associated with neuronal damage in other neurological diseases [73–76]. Nevertheless, microglia activity is also known to influence neuronal activity and survival in the injured brain [77] as well as to facilitate synaptic organization and brain repair [75]. Future studies using this model would benefit from measuring HIV viral protein levels (e.g. gp120, tat, vpr) for both, HIV-1_JR − CSF_ and HIV-1_CH040_ infection, to examine whether differential expression of viral proteins contribute to strain dependent effects on CNS pathology.

An important issue to consider regarding this study is that the brain of BLT humanized NSG mice is not known to be repopulated with human microglia, which are an important target of HIV infection in the brain. Further, intrinsic to most humanized mouse models, we evaluated the effects on CNS pathology on murine cells instead of human cells, which poses a limitation in directly translating the data to the respective human cells. However, it is quite remarkable that even under these circumstances, the mouse cells responded to HIV infection in a manner that is consistent with what would be expected from human cells. Therefore, our observations validate this model for the in vivo analysis of the effect of HIV on CNS pathogenesis. Future studies will be needed in order to characterize CNS pathology in more detail by for example including other quantification methods, such as Western blot analyses for assessment of viral proteins, proinflammatory markers (e.g. inflammasome, IL-6, TNF-α) in relation to neuroinflammation as measured by astrogliosis and microgliosis, and pyroptosis markers (e.g. IL-1β, IL-18, caspase-1) in relation to neuronal loss/dendritic injury. Another important issue to consider regarding the current study is the difficulty in studying viral evolution throughout the course of infection due to the short lifespan of mice compared to humans, and therefore, our results might be more representative of acute infection. We did not assess CNS pathology in the presence of ART. ART has been shown to significantly decrease CNS pathology in PLWH and animal models [34, 39, 78–80]. Nevertheless, in virally suppressed PLWH viral load has been detected in the CSF and use of post-mortem tissue has confirmed HIV DNA/RNA detection in the brain of PLWH under ART [7–12]. Consistent with these observations, we previously demonstrated that ART effectively decreases HIV RNA and DNA levels in the brain of infected humanized mice but it does not fully eliminate HIV infection from the brain, even if plasma viral loads were below the level of detection [34]. But even under these circumstances we were able to demonstrate that ART treatment results in a restoration of T cell homeostasis in the brain [34]. Future studies will evaluate to what extent CNS pathology can be reduced and/or reversed with ART treatment.

## Conclusion

In the present study, we evaluated the impact of two HIV-1 strains on CNS pathology. Although both viral strains induced neuronal injury and astrogliosis in all five CNS regions assessed, infection with HIV-1_JR − CSF_, a T cell tropic strain, predominantly affected neuronal health, whereas infection with HIV-1_CH040_ [33, 69], a macrophage tropic strain, had more prominent effects on neuroinflammation. These results suggest that macrophage infection may contribute to a heightened inflammatory state in the brain, specifically during acute CNS infection. The sustained effect of infection with HIV-1_JR − CSF_ suggests long-term induced CNS consequences and is a compelling area for further investigation in light of recent findings demonstrating subtypes of T cells that resist HIV-mediated cytopathy [22, 30]. These results further support the early initiation of ART to minimize the long-term neurological damage of HIV infection on the CNS.

### Electronic supplementary material

Below is the link to the electronic supplementary material.

Supplementary Material 1

## Data Availability

The datasets used and/or analyzed during the current study are available from the corresponding author on reasonable request.

## Abbreviations

ANOVA: Analysis of variance
ART: Antiretroviral therapy
BBB: Blood-brain-barrier
CCR5: C-C chemokine receptor type 5
CFS: Cerebrospinal fluid
CNS: Central nervous system
GFAP: Glial fibrillary acidic protein
HAND: HIV-associated neurocognitive disorders
HIV: Human immunodeficiency virus
HIV-1_ADA_: Laboratory derived macrophage-tropic virus
HIV-1_CH040_: CCR5-using (R5) macrophage-tropic HIV strain
HIV-1_JR − CSF_: CCR5-using (R5) T-tropic HIV strain, early-passage strain
HIVE: HIV encephalitis
Iba-1: Ionized calcium-binding adapter molecule 1
MAP2ab: Microtubule-associated protein 2, ab
NeuN: Neuronal nuclear
PBMC: Peripheral blood mononuclear cell
PLWH: People living with HIV
SEM: Standard error of the mean
°C: Degrees Celsius
