# Supplementary material for: In situ analysis of neuronal injury and neuroinflammation during HIV-1 infection
Source: Retrovirology. 2024 Jul 1;21:11. doi: 10.1186/s12977-024-00644-z (PMC11215835; doi:10.1186/s12977-024-00644-z)
Supplement: Supplementary file 1 — Supplementary Material 1 [file 12977_2024_644_MOESM1_ESM.pdf]

In situ analysis of neuronal injury and neuroinflammation during HIV-1 infection

Jenna B. Honeycutt[1#], Angela Wahl[1,2#], Jacob K. Files[2], Alexis F. League[3], Barkha J. Yadav-Samudrala[3], J. Victor Garcia[1,2*], Sylvia Fitting[3*]

[1]Division of Infectious Diseases, Center for AIDS Research, University of North Carolina at Chapel Hill, School of Medicine, Chapel Hill, North Carolina, 27599, USA; [2]Department of Microbiology, University of Alabama at Birmingham, Birmingham, AL, 35294, USA; [3]Department of Psychology & Neuroscience, University of North Carolina at Chapel Hill, Chapel Hill, North Carolina, 27599, USA

[#]These authors contributed equally to this work

***Corresponding Authors:**

Sylvia Fitting, Ph.D.

Department of Psychology & Neuroscience

University of North Carolina at Chapel Hill

235 E. Cameron Ave., Davie 126

Chapel Hill, North Carolina, 27599, USA

Office: +1-919-962-6595

E-mail: sfitting@email.unc.edu

J. Victor Garcia, Ph.D. CPC

Department of Microbiology

University of Alabama at Birmingham

845 19th Street South

Birmingham, Alabama, 35294, USA

Office: +1-205-934-1980

E-mail: jvgarcia@uab.edu

Supplemental Figures

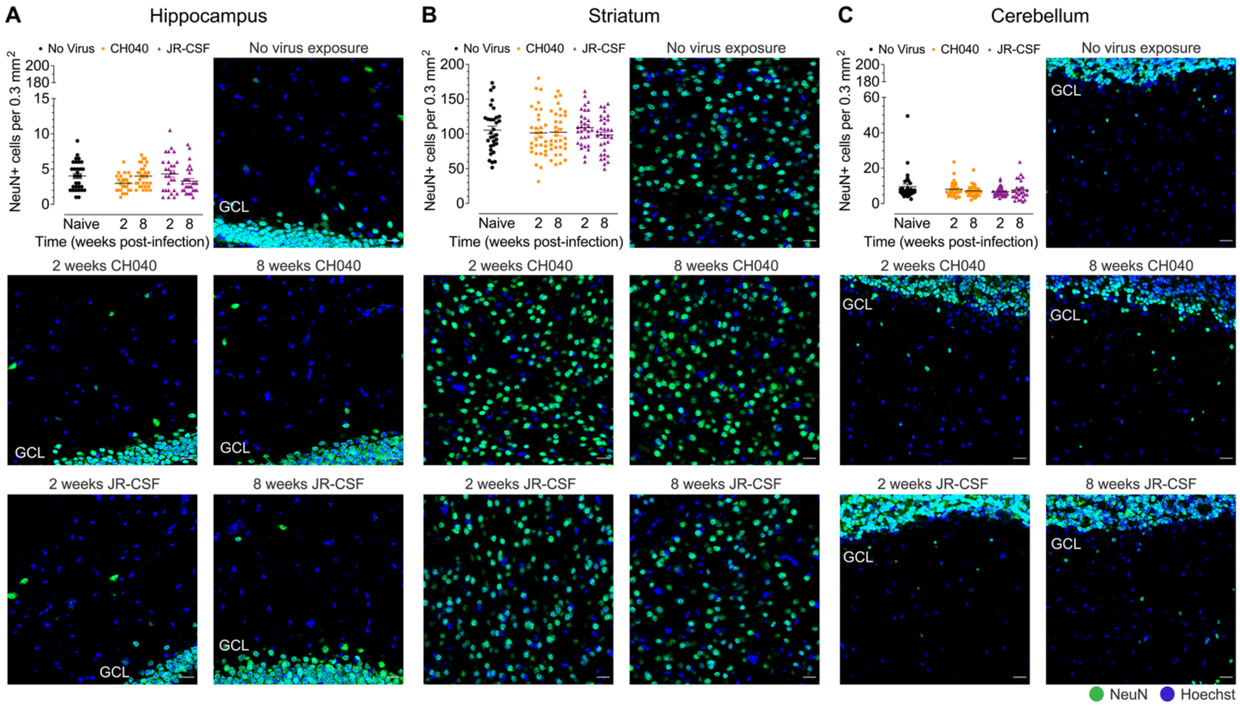

Figure S1. HIV infection induces no neuronal loss in the hippocampus, striatum, and cerebellum. Quantification of NeuN$^+$ neurons with representative images of NeuN$^+$ neurons (green, Hoechst/cell nuclei in blue) for all five BLT humanized mouse groups in the hippocampus **(A)**, striatum **(B)**, and cerebellum **(C)**. Statistical significance was assessed by one-way ANOVAs. Sample derived from 5-8 sections per mouse with *n* = 4 mice per group. NeuN: neuronal nuclear protein. Scale bars = 20 µm.

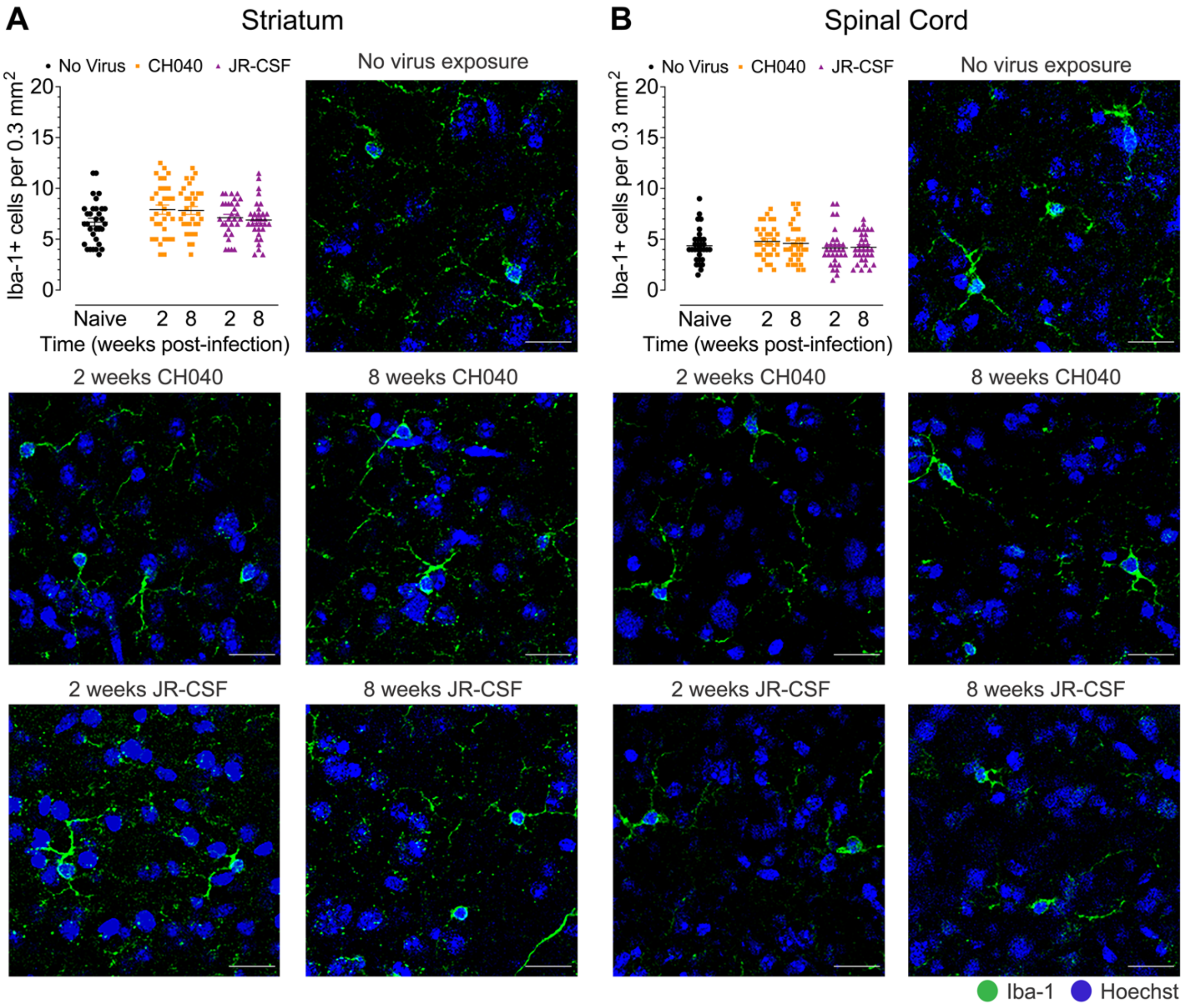

Figure S2. HIV infection induces no microglia activation in the striatum and spinal cord. Quantification of Iba-1^{+} microglia with representative images at higher magnification of Iba-1^{+} microglial (green, Hoechst/cell nuclei in blue) for all five BLT humanized mouse groups in the striatum **(A)**, and spinal cord **(B)**. Statistical significance was assessed by one-way ANOVAs. Sample derived from 5-8 sections per mouse with *n* = 4 mice per group. Iba-1: ionized calcium binding adaptor molecule 1. Scale bars = 20 μm.

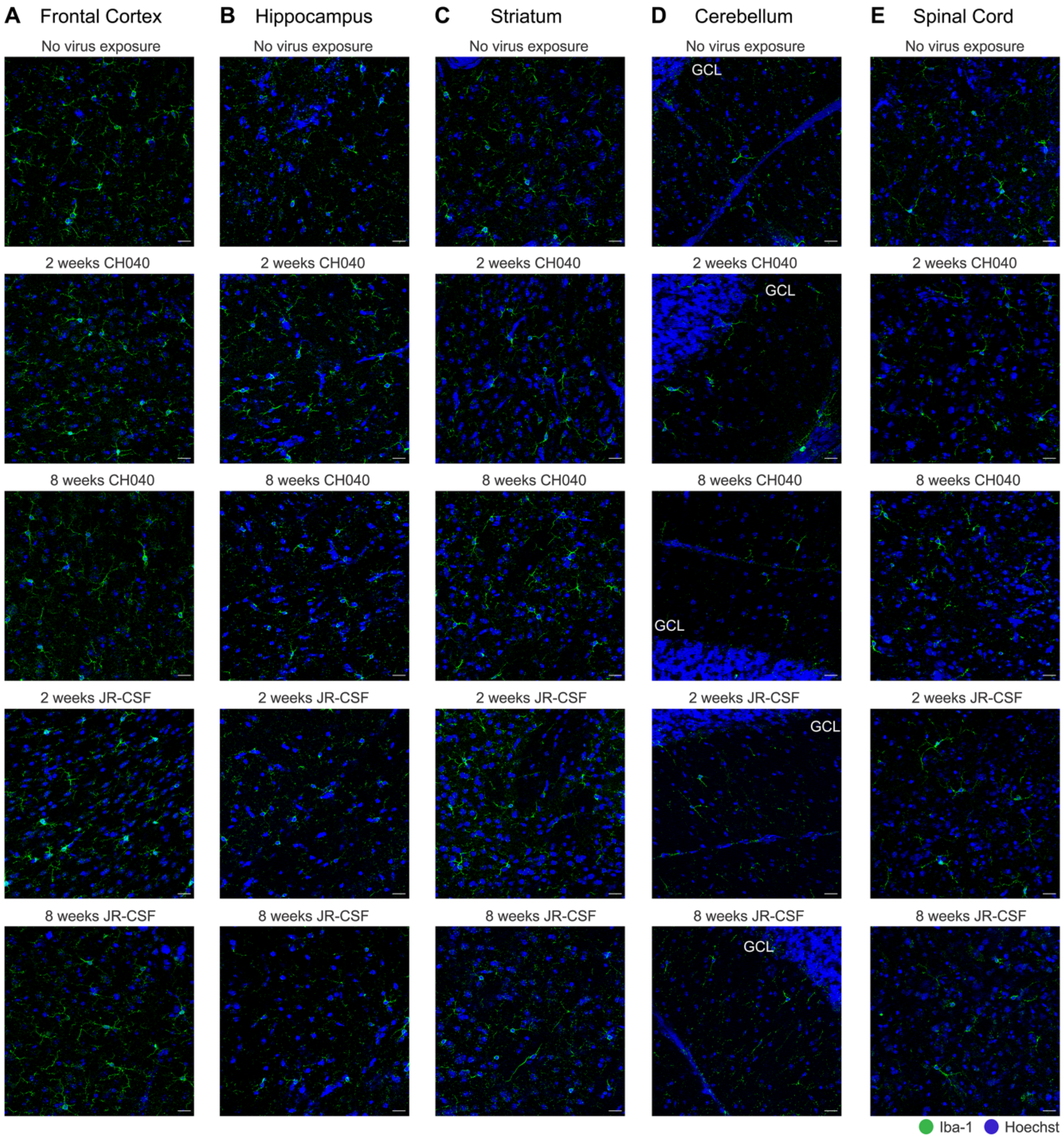

Figure S3. Microglial activation is CNS region specific and more prominent in HIV-1$_{CH040}$ infected mice. Quantification of Iba-1^{+} microglia with representative images at original magnification of Iba-1^{+} microglial (green, Hoechst/cell nuclei in blue) for all five BLT humanized mouse groups in the frontal cortex **(A)**, hippocampus **(B)**, striatum **(C)**, cerebellum **(D)**, and spinal cord **(E)**. Statistical significance was assessed by one-way ANOVAs. Sample derived from 5-8 sections per mouse with *n* = 4 mice per group. Iba-1: ionized calcium binding adaptor molecule 1. Scale bars = 20 μm.
